# The PIEZO1/CISD1/IP3R Mechanotransduction Axis Drives Macrophage Mitochondrial Dysfunction and Inflammatory Activation in Pulmonary Fibrosis

**DOI:** 10.1002/mco2.71080

**Published:** 2026-10-15

**Authors:** Yungeng Wei, Chao Xie, Xiangsheng Yang, Xiao Xiao Tang

**Affiliations:** ^1^ State Key Laboratory of Respiratory Disease National Clinical Research Center for Respiratory Disease National Center for Respiratory Medicine Guangzhou Institute of Respiratory Health The First Affiliated Hospital of Guangzhou Medical University Guangzhou China; ^2^ Guangzhou National Laboratory Guangzhou China; ^3^ GMU‐GIBH Joint School of Life Sciences Guangzhou Medical University Guangzhou China

**Keywords:** CISD1, idiopathic pulmonary fibrosis, macrophage, mitochondria, PIEZO1

## Abstract

Idiopathic pulmonary fibrosis (IPF) is a progressive and fatal interstitial lung disease characterized by excessive extracellular matrix (ECM) deposition and elevated tissue stiffness. While aberrant mechanotransduction is increasingly recognized as a key driver of fibrotic progression, the mechanisms by which mechanical cues orchestrate macrophage dysfunction remain poorly elucidated. Here, we identify the mechanosensitive ion channel PIEZO1 as a key mediator coupling matrix stiffening to macrophage inflammatory activation in pulmonary fibrosis. We demonstrate that PIEZO1 is substantially upregulated in fibrotic lungs, and that its activation drives intracellular calcium overload via the CISD1/IP3R signaling axis. This process elicits aberrant opening of the mitochondrial permeability transition pore (MPTP), causing mitochondrial dysfunction and augmented pro‐inflammatory macrophage polarization. Conversely, PIEZO1 inhibition suppresses the CISD1/IP3R signaling cascade, alleviates calcium dysregulation, prevents mitochondrial damage, and attenuates inflammatory activation. In vitro CISD1 inhibition ameliorates PIEZO1‐driven mitochondrial abnormalities and pro‐inflammatory signaling in macrophages. Furthermore, genetic knockdown of CISD1 mitigates mitochondrial abnormalities and attenuates bleomycin (BLM)‐induced lung fibrosis in mice. Collectively, these findings identify a previously unrecognized PIEZO1/CISD1/IP3R mechanotransduction axis coupling tissue stiffening to macrophage‐mediated inflammation and fibrosis. This study offers mechanistic insights into fibrosis‐associated immune dysregulation, highlighting PIEZO1 and CISD1 as promising therapeutic targets for pulmonary fibrosis.

## Graphical abstract

In the pulmonary fibrotic microenvironment, mechanical signals activate PIEZO1 to mediate Ca^2+^ entry into macrophages. Increased CISD1/IP3R activity promotes Ca^2+^ transfer from the endoplasmic reticulum to mitochondria, leading to mitochondrial Ca^2+^ overload and opening of the mitochondrial permeability transition pore (MPTP). Consequently, macrophages exhibit enhanced profibrotic capacity. Graphical abstract was created with BioRender.com.

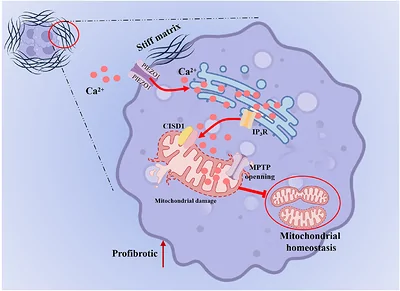

## 1 Introduction

Idiopathic pulmonary fibrosis (IPF) is a fatal interstitial lung disease characterized by progressive scarring of lung parenchyma, with a median survival of 2–3 years post‐diagnosis and limited therapeutic options [1, 2, 3]. In the progression of IPF, excessive deposition of extracellular matrix (ECM) in the pulmonary interstitium increases the tissue stiffness, altering the mechanical stress between the matrix and cells [4]. Increased stiffness of ECM exerts various effects on multiple cell phenotypes and the tissue microenvironment, including promoting release of transforming growth factor‐β1 (TGF‐β1) from the ECM [5], facilitating differentiation of fibroblasts into myofibroblasts by activating focal adhesion kinase (FAK) [6]. In addition, macrophages are more dispersed and flattened on rigid substrates, with formation of podosomes on the cell surface [7, 8]. These observations collectively suggest that the mechanical signals generated by increased ECM stiffness are associated with fibrotic diseases, yet the role of these mechanical signals in pulmonary fibrosis remains incompletely understood.

The changes in cell behavior caused by increased ECM stiffness‐generated mechanical stress usually depend on mechanosensitive ion channels, which initiate membrane depolarization in excitable cells or trigger calcium signals in non‐excitable cells [9], thereby activating downstream signaling pathways and leading to changes in cell phenotypes and behaviors [10]. Among these channels, PIEZO1, a new mechanosensitive calcium channel, evolutionarily conserved alongside PIEZO2 [11], is crucial for sensing and transducing various mechanical stimuli [12]. Studies have shown upregulated expression of PIEZO1 in lung tissues with acute respiratory distress syndrome (ARDS)‐associated pulmonary fibrosis and it exacerbates fibrotic progression [13]. Additionally, PIEZO1 is involved in fibrosis development in multiple organs [14, 15, 16], indicating its critical role in fibrotic diseases. Despite this, how PIEZO1‐mediated mechanotransduction contributes specifically to IPF pathogenesis remains underexplored.

Macrophages serve as key orchestrators of fibrosis [3, 17] and can also respond to mechanical signals in the extracellular microenvironment, including changes in macrophage chemotaxis, polarization, and phagocytic function [8, 18, 19]. While immune dysregulation, particularly macrophage‐mediated inflammation, is crucial to IPF pathogenesis, the role of mechanical stress in driving macrophage activation remains understudied. Emerging evidence highlights that mechanical stimuli—such as increased tissue stiffness in fibrotic tissue—activate mechanosensitive ion channels, including PIEZO1, to modulate immune responses [20, 21]. Studies reveal that PIEZO1 mediates calcium influx in macrophages upon mechanical stress, triggering downstream inflammatory cascades [20, 21]. Notably, PIEZO1 activation in macrophages has been linked to metabolic reprogramming and enhanced efferocytosis [20], yet its role in mitochondrial homeostasis during fibrosis remains elusive. Mitochondrial dysfunction and impaired homeostasis play an important role in the process of pulmonary fibrosis [17]. Intriguingly, mitochondrial calcium overload, regulated by the mitochondrial‐associated endoplasmic reticulum membrane (MAM) proteins such as CDGSH iron‐sulfur domain‐containing protein 1 (CISD1, also known as mitoNEET) and inositol 1,4,5‐trisphosphate receptor (IP3R), is implicated in mitochondrial permeability transition pore (MPTP) activation and subsequent oxidative stress [22, 23]. Dysregulation of MAMs disrupts Ca^2^^+^ flux and ATP synthesis, exacerbating cellular damage—a phenomenon observed in neurodegenerative diseases but scarcely studied in fibrotic contexts [24]. Given these findings, we hypothesized that mechanical signals may regulate intracellular calcium ion homeostasis and mitochondrial function through the PIEZO1/CISD1 pathway, and thereby contribute to the pathogenesis of IPF.

In this study, we found upregulated expression of PIEZO1 and CISD1 in the lungs of mice with BLM‐induced fibrosis and patients with pulmonary fibrosis. Knockdown of CISD1 in the mouse lung via shRNA ameliorated BLM‐induced pulmonary fibrosis. In vitro, we explored the effect of matrix stiffness on macrophage mitochondrial homeostasis and polarization, and further investigated the underlying molecular mechanisms. In pulmonary alveolar macrophages (AMs), pathologically increased extracellular matrix stiffness can lead to intracellular‌ calcium overload, mitochondrial dysfunction, and pro‐inflammatory M1 polarization. Mechanistically, elavated matrix stiffening in AMs upregulates PIEZO1 and CISD1 expression, promotes endoplasmic reticulum (ER) calcium release through IP3R activation, and induces intracellular calcium overload and subsequent mitochondrial dysfunction, ultimately facilitating M1 polarization. Either genetic knockdown or pharmacological inhibition of PIEZO1 or CISD1 ameliorates matrix stiffness‐induced mitochondrial dysfunction and M1 polarization in AMs. Collectively, our findings identify the PIEZO1/CISD1/IP3R axis as a critical regulator of matrix stiffness‐induced mitochondrial dysfunction and aberrant polarization in AMs, offering novel mechanistic insights and potential new therapeutic strategies for pulmonary fibrosis.

## 2 Results

### 2.1 Matrix Stiffness in Lung Fibrosis Activates Macrophage PIEZO1 Signaling

Mechanical stress signals induced by ECM stiffening are involved in various fibrotic diseases [25]. To determine whether mechanical stress‐induced PIEZO1 activation occurs in macrophages during pulmonary fibrosis, we detected the expression of PIEZO1 in macrophages from lung tissue of IPF patients and healthy controls (HCs) by immunofluorescence staining. We found increased PIEZO1 expression in lung macrophages from IPF patients (Figure 1A). Similarly, PIEZO1 expression was also elevated in macrophages from BLM‐induced pulmonary fibrosis mice (Figure 1B). Notably, primary AMs isolated from these BLM‐instilled mice showed increased PIEZO1 expression (Figure 1C). Macrophages were cultured in vitro using matrix gels of varying hardness to mimic the extracellular matrix stiffness in a fibrotic microenvironment. As the stiffness of the matrix gel increased, the expression of PIEZO1 in macrophages also increased (Figure 1D,E). These data indicate that during pulmonary fibrosis, macrophages can sense mechanical stress signals from the hardened ECM through the mechanosensor PIEZO1.

**FIGURE 1:**
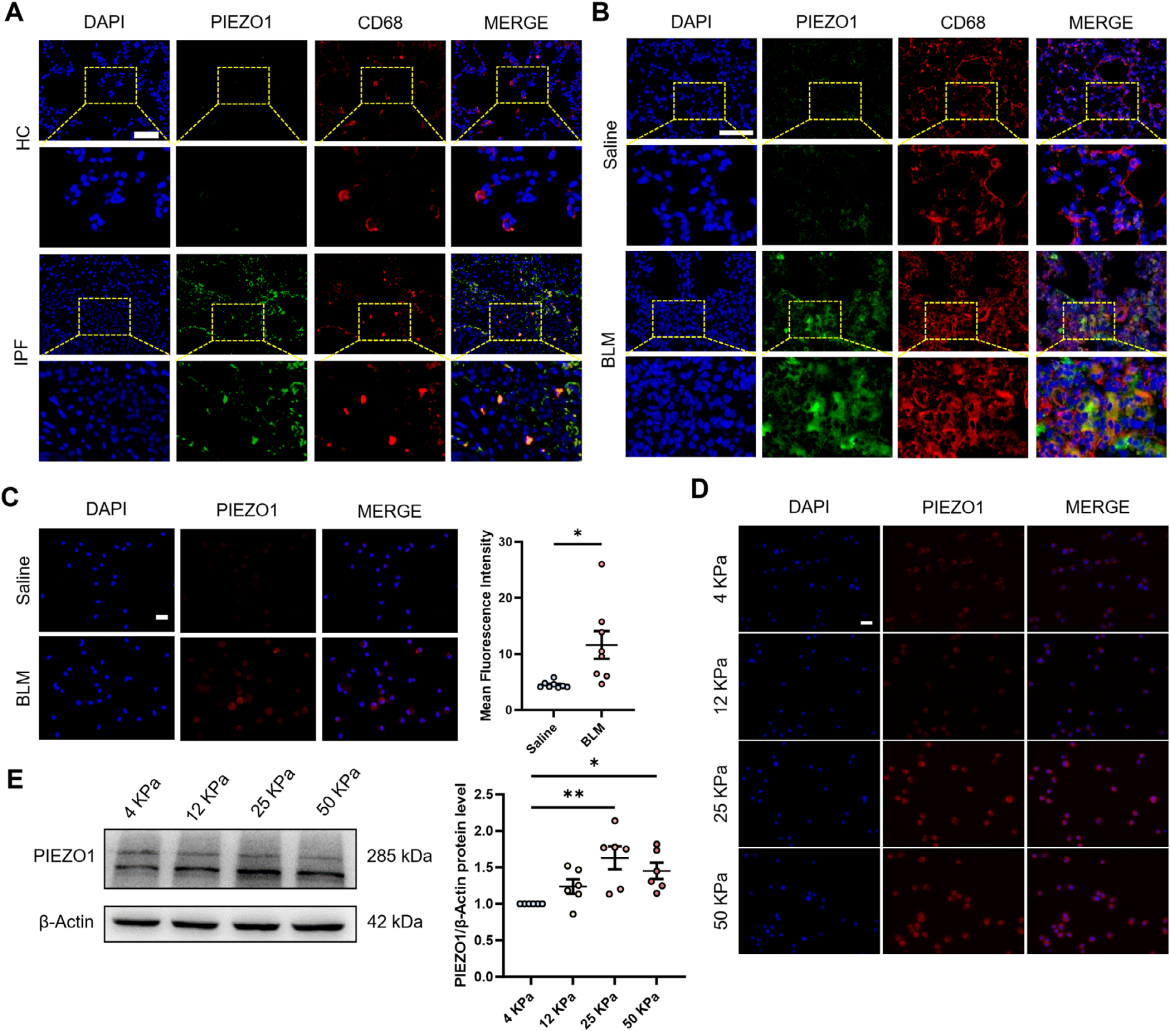
Macrophage PIEZO1 is activated under increased fibrotic stiffness. (A). Immunofluorescence detection of CD68 (red) and PIEZO1 (green) co‐localization in lung tissues of IPF patients, scale bar = 100 µm, *n* = 3 biological replicates. (B). Immunofluorescence detection of CD68 (red), PIEZO1 (green) co‐localization in lung tissues of bleomycin‐induced fibrotic mice, scale bar = 100 µm. (C) Immunofluorescence staining detection of PIEZO1 expression in primary macrophages in BLM‐induced mouse alveolar lavage fluid, scale bar = 20 µm. (D). Immunofluorescence detection of PIEZO1 expression in MH‐S cells cultured on different stiffness matrix gels for 24 h, scale bar = 20 µm. (E). WB detection and quantitative analysis of PIEZO1 expression in MH‐S cells after culturing on different stiffness matrix gels for 24 h, *n* = 6 biological replicates.

### 2.2 Matrix Stiffness Activates MPTP Opening, Leading to Mitochondrial Damage

We previously reported that changes in macrophage mitochondrial function play a role in pulmonary fibrosis [3, 17]. Here, we found that stiffness stimulation caused depolarization of the mitochondrial membrane potential (MMP) in macrophages (Figure 2A) and increased mitochondrial reactive oxygen species (mtROS) (Figure 2B), indicating that mechanical signal induces mitochondrial damage in AMs. MPTP opening, one of the important indicators of mitochondrial damage, is usually caused by mitochondrial calcium overload. We showed that macrophages cultured on stiff matrix exhibited increased concentrations of calcium and sodium ions (Figure 2C and Figure S1), as well as an increased opening of MPTP (Figure 2D). To further confirm that mechanical stress induced MPTP opening, we examined the gene and protein expression of key proteins mediating MPTP and found that mechanical stress induced an increase in cyclophilin D (CypD, also known as peptidyl‐prolyl cis‐trans isomerase F) but had little effect on adenine nucleotide translocator 1 (ANT1) expression (Figure 2E,F). We also examined markers of macrophage polarization and found that matrix stiffness promoted the polarization of AMs toward a proinflammatory phenotype, as evidenced by the increased expression of inducible nitric oxide synthase (iNOS) and CD86, qPCR results also confirmed this finding (Figure 2G, Figure S2). Yet the changes of CD163 and CD206, markers of M2 polarization, were not significantly different. These results indicated that matrix stiffness induced mitochondrial damage in AMs and triggered pro‐inflammatory phenotypic polarization.

**FIGURE 2:**
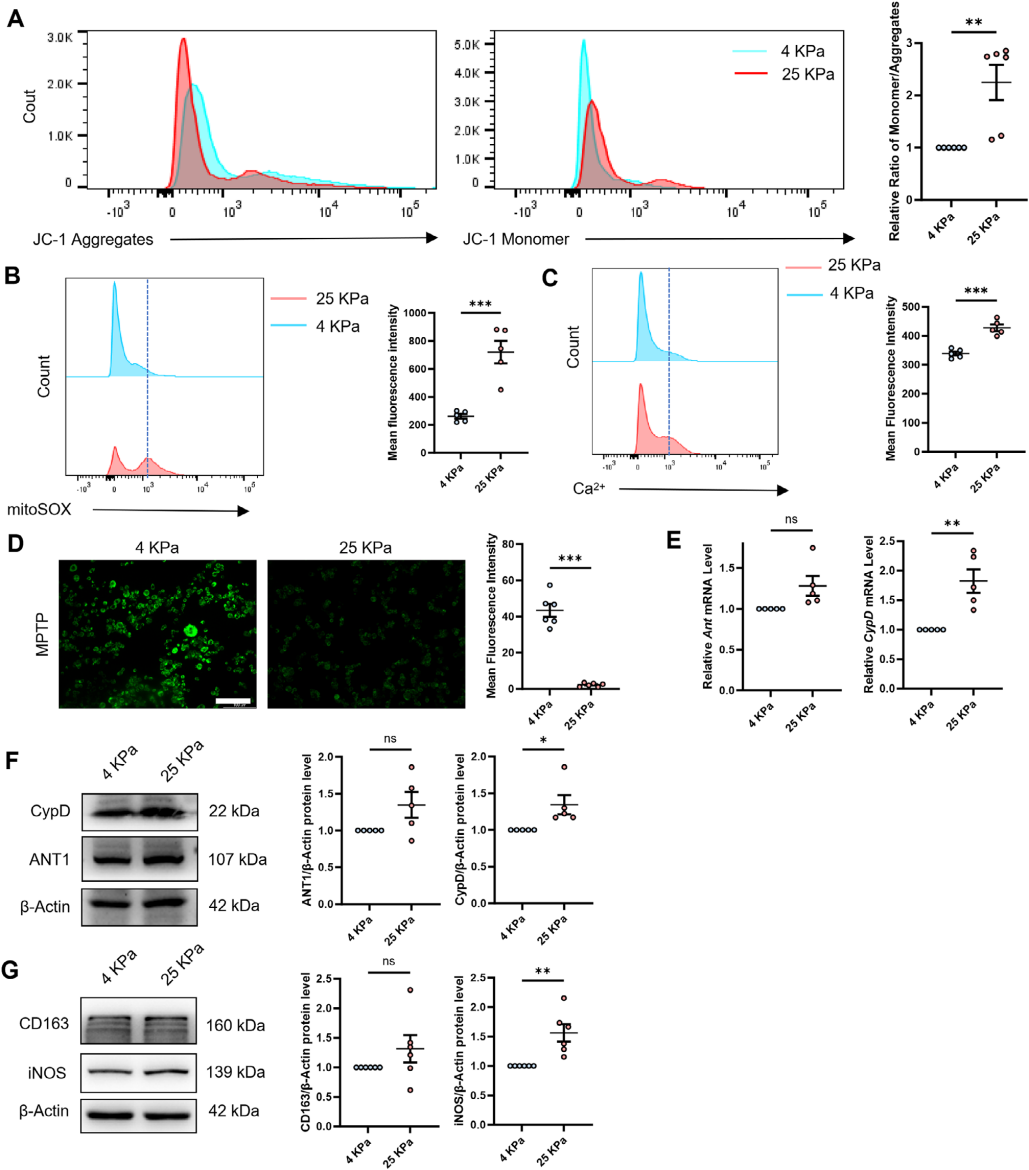
Increased mechanical stiffness induces MPTP opening and mitochondrial damage in macrophages. (A) MH‐S cells were incubated with different stiffness matrix gels for 24 h. Mitochondrial membrane potential was labeled using the mitochondrial membrane potential probe JC‐1 and detected by flow cytometry, *n* = 6 biological replicates. (B). MH‐S cells were incubated with different stiffness matrix gels for 24 h. Mitochondrial reactive oxygen species content in cells was measured by using the mitoSOX probe and detected by flow cytometry, *n* = 5 biological replicates. (C). MH‐S cells were incubated with different stiffness matrix gels for 24 h. Calcium ion concentration in cytoplasm was detected using flow cytometry, *n* = 5 biological replicates. (D). MH‐S cells were incubated with different stiffness matrix gels for 24 h. MPTP activity of macrophages after matrix gel culture was assayed using the MPTP kit, scale bar = 100 µm, *n* = 6 biological replicates, stronger green fluorescence corresponds to reduced MPTP opening and preserved mitochondrial integrity, while weaker fluorescence indicates elevated MPTP opening and impaired mitochondrial structural integrity. (E). MH‐S cells were incubated with different stiffness matrix gels for 24 h. Expression of mitochondrial membrane permeability transition pore‐associated genes ANT1 and CypD detected by qPCR, *n* = 5 biological replicates. (F). MH‐S cells were incubated with different stiffness matrix gels for 24 h. Expression of ANT1 and CypD was detected by WB, *n* = 5 biological replicates. (G). MH‐S cells were incubated with different stiffness matrix gels for 24 h. Expression of CD163 and iNOS was detected by WB, *n* = 6 biological replicates.

### 2.3 Matrix Stiffness Mediates ER Calcium Release to Activate MPTP via CISD1

Mitochondrial membrane protein CISD1 can affect mitochondrial function by regulating intracellular calcium ion homeostasis [26]. We found an increased CISD1 expression in lung macrophages from IPF patients (Figure 3A). Mice with BLM‐induced pulmonary fibrosis displayed increased CISD1 expression in macrophages from lung tissue sections (Figure 3B), a phenotype that was recapitulated in primary AMs (Figure 3C). Moreover, enhanced expression of CISD1 and its downstream IP3R was also observed in AMs cultured using stiffness matrix gel (Figure 3D), indicating that mechanical signals can enhance expression of CISD1/IP3R in AMs during pulmonary fibrosis.

**FIGURE 3:**
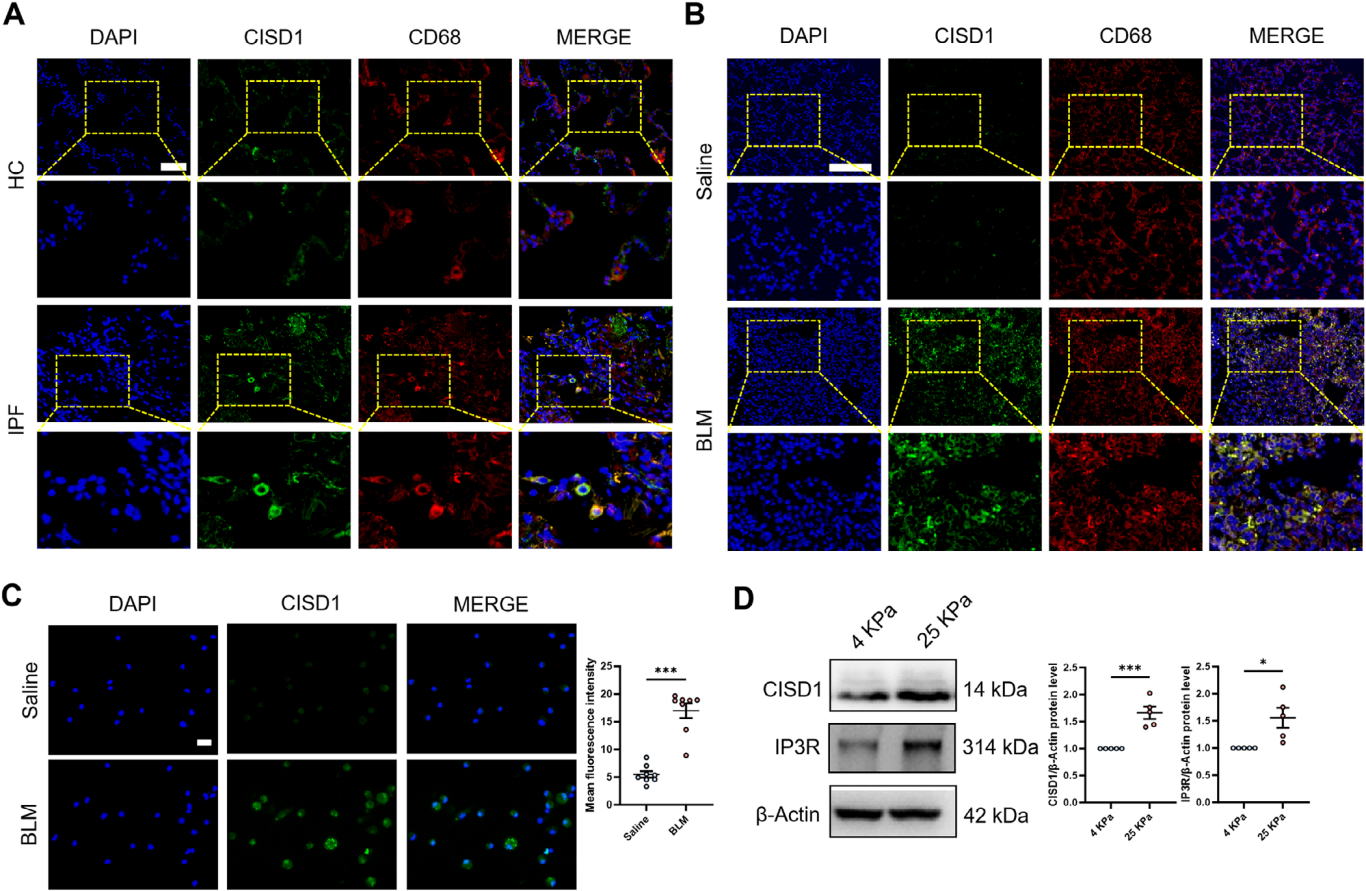
Increased mechanical stiffness activates calcium ion channels in macrophages. (A). Immunofluorescence staining to detect CISD1 (green) expression in macrophages (CD68, red) of lung sections from IPF patients. Scale bar = 100 µm, *n* = 3 biological replicates. (B). Immunofluorescence staining to detect CISD1 (green) expression in macrophages (CD68, red) of BLM‐induced mouse lung sections. Scale bar = 100 µm. (C). Immunofluorescence staining to detect CISD1 expression in primary macrophages in BLM‐induced mouse alveolar lavage fluid. Scale bar = 20 µm. (D). MH‐S cells were cultured with different stiffness matrix gels for 24 h, and the expression of CISD1 and IP3R was detected by WB, *n* = 5 biological replicates.

### 2.4 Inhibition of CISD1 Alleviates Mitochondrial Damage via Suppressing MPTP Opening

To explore the regulatory role of CISD1 in calcium ion and mitochondrial homeostasis, we generated CISD1‐knockdown AMs and found that CISD1 knockdown reduced matrix stiffness‐enhanced calcium ion concentration (Figure 4A) as well as the opening of MPTP (Figure 4B). Additionally, we detected changes in mtROS and MMP. As expected, CISD1 knockdown suppressed the upregulation of mtROS triggered by mechanical stress (Figure 4C) and MMP depolarization (Figure 4D,E). Meanwhile, knockdown of CISD1 downregulated the expression levels of IP3R and CypD, further substantiating the regulatory role of CISD1 in calcium homeostasis and MPTP opening. Consistently, CISD1 knockdown decreased the expression of NLRP3 and iNOS, confirming that genetic suppression of CISD1 mitigates mechanical stress‐induced inflammatory activation of AMs (Figure 4F,G). We also applied CISD1‐specific inhibitor NL‐1 to inhibit CISD1 activity, and in line with the results of CISD1 knockdown, NL‐1 treatment effectively blunted the mechanical stress‐elicited rise in calcium ion concentration (Figure S3A) and MPTP opening (Figure S3B). Assessments of mtROS and MMP further uncovered that NL‐1 intervention counteracted mechanical stress‐evoked mitochondrial damage (Figure S3C–E). NL‐1 exposure also dampened the mechanical stress‐augmented abundance of IP3R, MUC, and CypD, as well as NOD‐like receptor thermal protein domain associated protein 3 (NLRP3) and iNOS (Figure S3F,G). Cumulatively, these findings establish that matrix stiffness elicits mitochondrial impairment and inflammatory activation of macrophages through CISD1.

**FIGURE 4:**
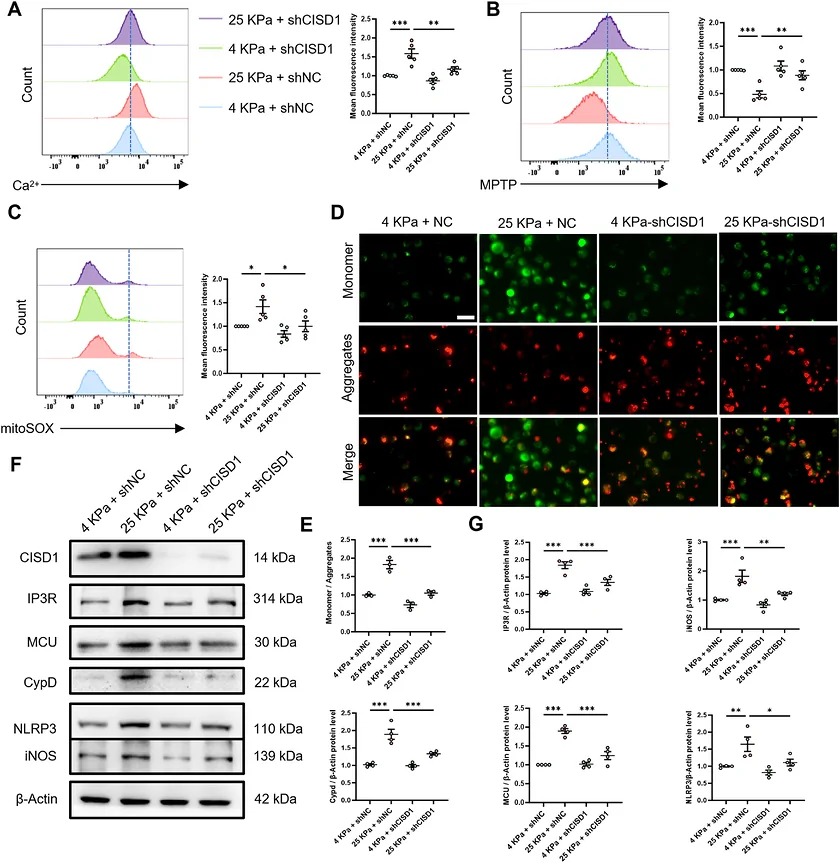
Knockdown of CISD1 attenuates mechanical stiffness‐mediated macrophage MPTP opening and mitochondrial damage. (A). MH‐S cells with knockdown of CISD1 were cultured on different stiffness matrix gels, and cytosolic calcium ions were detected by flow cytometry, *n* = 5 biological replicates. (B). MH‐S cells with knockdown of CISD1 were cultured on different stiffness matrix gels, and MPTP activity was detected by flow cytometry, *n* = 5 biological replicates. (C). MH‐S cells with knockdown of CISD1 were cultured on different stiffness matrix gels, mitochondrial reactive oxygen species content was detected by flow cytometry, *n* = 5 biological replicates. (D and E) MH‐S cells with knockdown of CISD1 were cultured on different stiffness matrix gels, mitochondrial membrane potential was detected by immunofluorescence microscope, *n* = 3 biological replicates, scale bar = 20 µm. (F and G). MH‐S cells with knockdown of CISD1 were cultured on different stiffness matrix gels, and MPTP‐related proteins and inflammation‐related proteins were detected by WB, *n* = 4 biological replicates.

### 2.5 Matrix Stiffness Modulates CISD1‐Mediated Mitochondrial Damage via PIEZO1

It remains unclear whether matrix stiffness regulation of CISD1 depends on PIEZO1. Here, we treated macrophages with Yoda1, a well‐validated pharmacological agonist of PIEZO1 (Figure S4), and observed that the expression levels of CISD1, IP3R, and CypD were upregulated in macrophages with PIEZO1 activation (Figure 5A). Yoda1 treatment also elevated mtROS levels in macrophages (Figure 5B), triggered MMP depolarization (Figure 5C), and promoted MPTP opening (Figure 5D), indicating that activation of PIEZO1 is sufficient to initiate activation of the CISD/IP3R signaling cascade and subsequently induce mitochondrial damage. In addition, we treated macrophages cultured on different stiffness substrates with GsMTx4, a selective and widely used PIEZO1 inhibitor, and found that inhibition of PIEZO1 attenuated the matrix stiffness‐induced elevation of CISD1, IP3R, and CypD (Figure 5E and Figure S5A). Mechanically triggered MPTP opening was also suppressed by PIEZO1 inhibition (Figure 5F and Figure S5B), alongside reduced mechanical stress‐induced mtROS overproduction and MMP depolarization (Figure 5G,H and Figure S5C,D). Consistent with these pharmacological results, siRNA knockdown of PIEZO1 effectively downregulated the expression of CISD1, IP3R, and CypD evoked by matrix stiffness (Figure S6A) and alleviated stiffness‐driven mitochondrial damage and MPTP opening (Figure S6B–D). These results confirmed that matrix stiffness activates the CISD1/IP3R pathway via PIEZO1, which mediates the subsequent mitochondrial damage.

**FIGURE 5:**
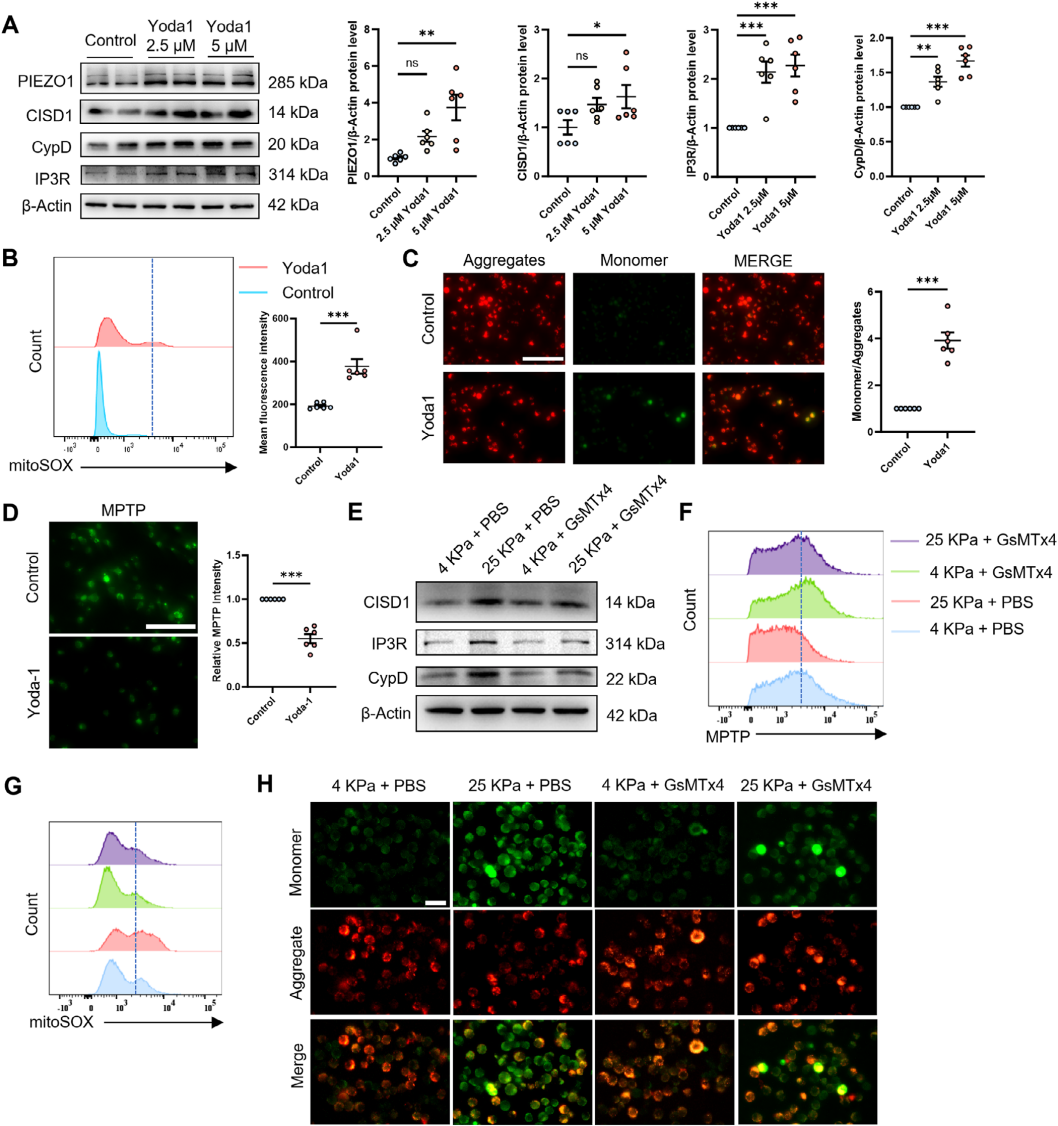
Matrix stiffness upregulates CISD1 via PIEZO1 to mediate mitochondrial damage. (A) MH‐S cells were treated with 2.5 µM or 5 µM Yoda1 for 24 h. WB assay was performed to detect the expression of MPTP‐related proteins, *n* = 6 biological replicates. (B) MH‐S cells were treated with 5 µM Yoda1 for 24 h, mitochondrial reactive oxygen species content was detected by flow cytometry, *n* = 6 biological replicates. (C) MH‐S cells were treated with 5 µM Yoda1 for 24 h, mitochondrial membrane potential was detected by immunofluorescence microscopy, *n* = 6 biological replicates, scale bar = 100 µm. (D) MH‐S cells were treated with 5 µM Yoda1 for 24 h, MPTP opening was detected by immunofluorescence microscopy, *n* = 6 biological replicates, scale bar = 100 µm. (E) MH‐S cells were cultured with matrix gels of different stiffness and simultaneously treated with PIEZO1 inhibitor GsMTx4, WB assay was performed to detect the expression of MPTP‐related proteins, *n* = 4 biological replicates. (F) MH‐S cells were cultured with different stiffness matrix gels and simultaneously treated with PIEZO1 inhibitor GsMTx4, and MPTP activity was detected by flow cytometry, *n* = 3 biological replicates. (G) MH‐S cells were cultured with different stiffness matrix gels and simultaneously treated with PIEZO1 inhibitor GsMTx4, mitochondrial reactive oxygen species content was detected by flow cytometry, *n* = 5 biological replicates. (H) MH‐S cells were cultured with different stiffness matrix gels and simultaneously treated with PIEZO1 inhibitor GsMTx4, mitochondrial membrane potential was detected by immunofluorescence microscopy, *n* = 4 biological replicates, scale bar = 20 µm.

### 2.6 Inhibition of IP3R Alleviates Matrix Stiffness‐Induced Mitochondrial Damage and MPTP Opening

To further verify the role of the CISD1/IP3R axis in mechanical stress‐induced mitochondrial homeostasis disruption, we used 2‐aminoethyl diphenylborinate (2‐APB) to inhibit IP3R activity. Inhibition of IP3R lowered intracellular calcium ion concentration (Figure 6A), and suppressed matrix stiffness‐induced MPTP opening (Figure 6B). Moreover, 2‐APB treatment also blunted matrix stiffness‐enhanced mtROS levels (Figure 6C) and alleviated MMP depolarization (Figure 6D). This lines of evidence indicate that IP3R inhibition mitigates matrix stiffness‐elicited mitochondrial dysfunction.

**FIGURE 6:**
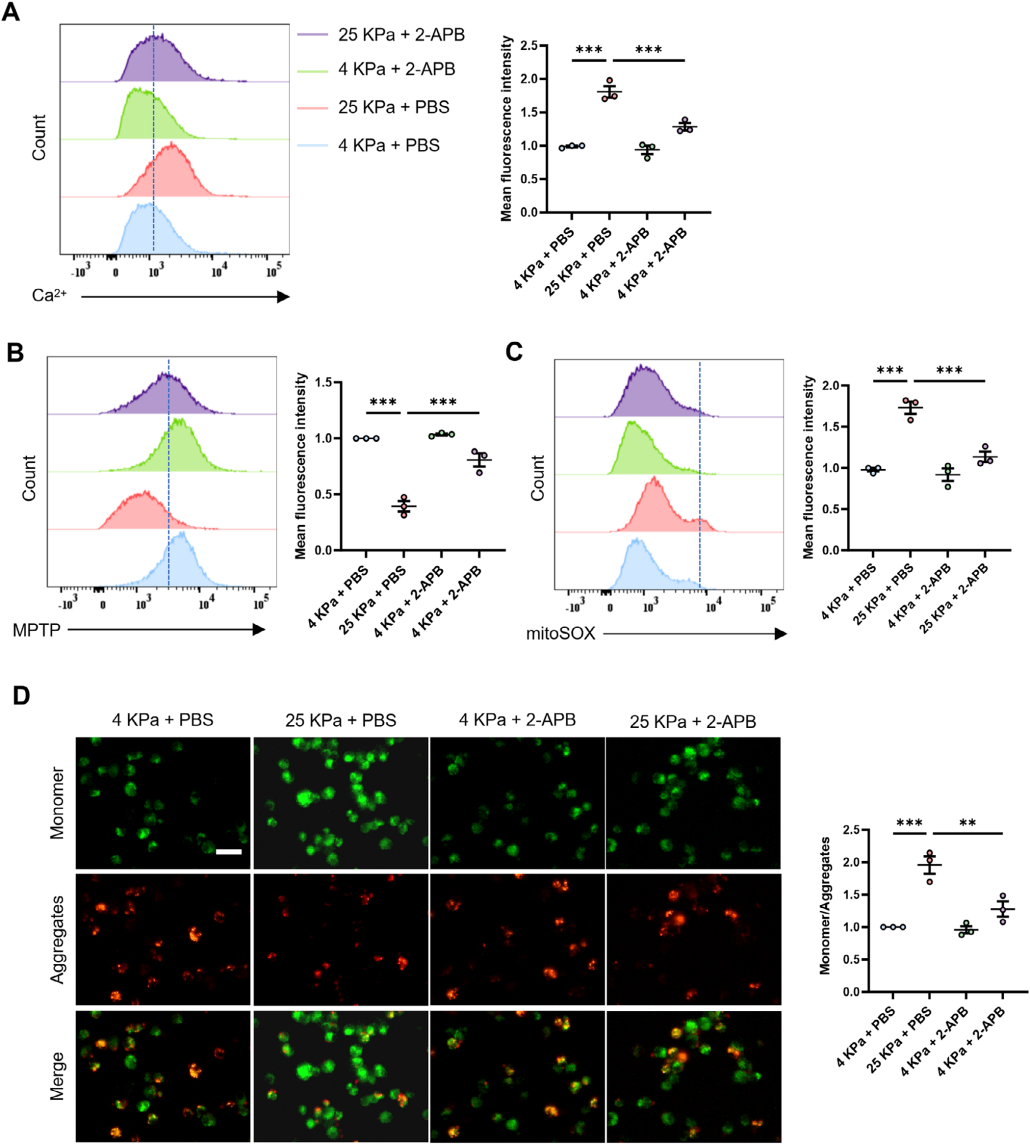
Matrix stiffness‐mediated mitochondrial damage depends on IP3R activity. (A) MH‐S cells were cultured with different stiffness matrix gels and simultaneously treated with IP3R inhibitor 2‐APB, and cytosolic calcium ions were detected by flow cytometry, *n* = 3 biological replicates. (B) MH‐S cells were cultured with different stiffness matrix gels and simultaneously treated with IP3R inhibitor 2‐APB, and MPTP activity was detected by flow cytometry, *n* = 3 biological replicates. (C) MH‐S cells were cultured with different stiffness matrix gels and simultaneously treated with IP3R inhibitor 2‐APB, mitochondrial reactive oxygen species content was detected by flow cytometry, *n* = 3 biological replicates. (D) MH‐S cells were cultured with different stiffness matrix gels and simultaneously treated with IP3R inhibitor 2‐APB, mitochondrial membrane potential was detected by immunofluorescence microscopy, *n* = 3 biological replicates, scale bar = 20 µm.

### 2.7 In vivo Knockdown of CISD1 Ameliorates the Function of Mitochondria and Macrophages in Fibrotic Mice

In vitro experiments have validated the regulatory role of the PIEZO1/CISD1/IP3R pathway in macrophage mitochondrial homeostasis. In a BLM‐induced mouse model of pulmonary fibrosis, we dissected the effects of this signaling pathway on macrophages via adeno‐associated virus (AAV)‐mediated CISD1 knockdown. A marked reduction in CypD expression was observed in BLM‐instilled mouse lung macrophages, demonstrating that MPTP opening was suppressed by CISD1 silencing (Figure 7A). Next, we applied flow cytometry to assess macrophage polarization, revealing that BLM instillation increased percentage of CD86^+^CD206^+^ macrophage subset with concurrent proinflammatory and profibrotic properties. By contrast, CISD1 knockdown markedly diminished the proportion of this dual‐positive macrophage subset, and abrogated the pro‐inflammatory and pro‐fibrotic capabilities of BLM‐primed lung macrophages (Figure 7B,C). We further profiled the dynamic alterations in lung tissue macrophage subpopulations. The proportion of interstitial macrophages (IMs) was elevated in BLM‐instilled lungs, whereas this proportion of IMs was reduced following CISD1 knockdown. Concurrently, CISD1 silencing also decreased the proportion of AMs while increasing the monocyte proportion, suggesting that CISD1 knockdown restrains recruitment of IMs and differentiation of bone marrow‐derived AMs (Figure 7D,E). Furthermore, we detected the expression of the M1 macrophage marker iNOS and the M2 marker CD206 by immunofluorescence staining, and the obtained results were consistent with those from flow cytometry analysis. The expression levels of iNOS and CD206 were both elevated in lung macrophages of BLM‐instilled mice, whereas CISD1 knockdown decreased their abundance (Figure 7F,G). This indicates that CISD1 knockdown restrains the aberrant macrophage polarization and recruitment, thereby reshaping the homeostasis of lung macrophages.

**FIGURE 7:**
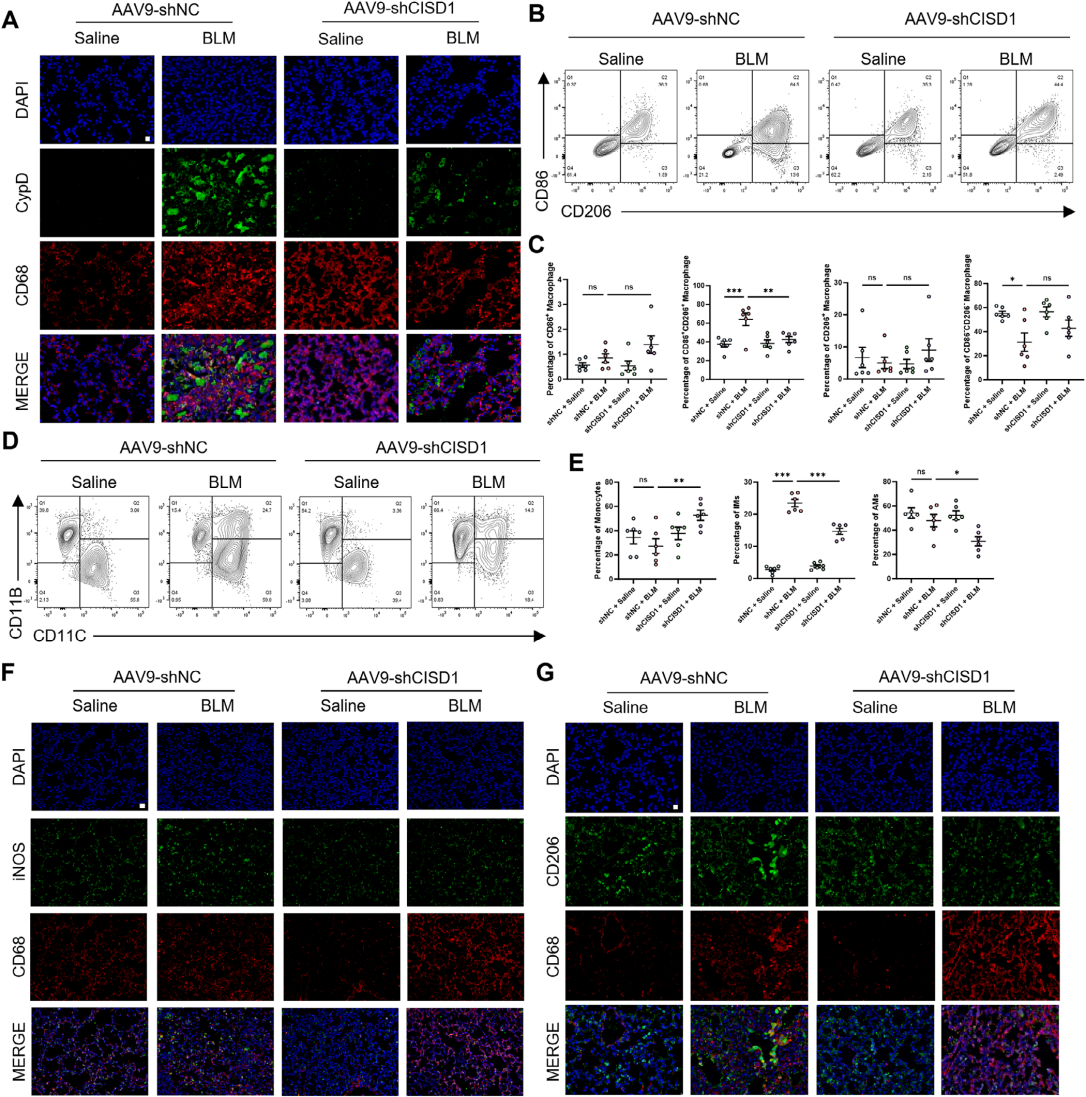
Knockdown of CISD1 improves BLM‐induced abnormal polarization of macrophages. (A) Following AAV9‐mediated knockdown of CISD1 in mice, pulmonary fibrosis was induced using BLM. Immunofluorescence staining was performed to detect expression of the MPTP protein CypD in pulmonary macrophages, scale bar = 20 µm. (B and C) Flow cytometry detection of polarization of pulmonary macrophages in each group, *n* = 6 biological replicates. (D and E). Flow cytometry detection of changes in the proportion of pulmonary macrophage subsets in each group of mice, *n* = 6 biological replicates. (F) Immunofluorescence staining detection of M1 polarization in pulmonary macrophages in each group of mice, scale bar = 20 µm. (G) Immunofluorescence staining detection of M2 polarization in pulmonary macrophages in each group of mice, scale bar = 20 µm.

### 2.8 In Vivo Knockdown of CISD1 Alleviates BLM‐Induced Pulmonary Fibrosis in Mice

To investigate the effects of CISD1 knockdown on BLM‐induced lung injury and pulmonary fibrosis, we assessed lung function across all experimental groups of mice using pulmonary function test (PFT) and found that CISD1 silencing mitigated BLM‐induced lung function decline (Figure 8A). Histopathological analysis via hematein–eosin (HE) and Masson's trichrome staining further corroborated that CISD1 knockdown alleviated BLM‐induced lung injury and fibrosis (Figure S7A,B). In vivo microCT analysis of mice further substantiated the beneficial effect of CISD1 knockdown on BLM‐induced pulmonary fibrosis, revealing a markedly elevated fraction of normally aerated lungs in CISD1‐silenced mice relative to the NC+BLM group (Figure 8B–D). Lastly, immunofluorescence staining for the myofibroblast marker α‐smooth muscle actin (α‐SMA) revealed that knockdown of CISD1 suppressed BLM‐driven myofibroblast activation (Figure S7C). The expression of the fibrosis‐related proteins fibronectin (FN) and collagen I (Col1) was enhanced in the lungs of BLM‐instilled mice, whereas CISD1 knockdown attenuated their upregulation (Figure 8E,F). As expected, CISD1 protein levels were also significantly reduced in the lungs of shCISD1‐treated mice, confirming the knockdown efficiency in vivo (Figure S8). These data collectively indicate that genetic ablation of CISD1 confers protection against BLM‐induced pulmonary fibrosis.

**FIGURE 8:**
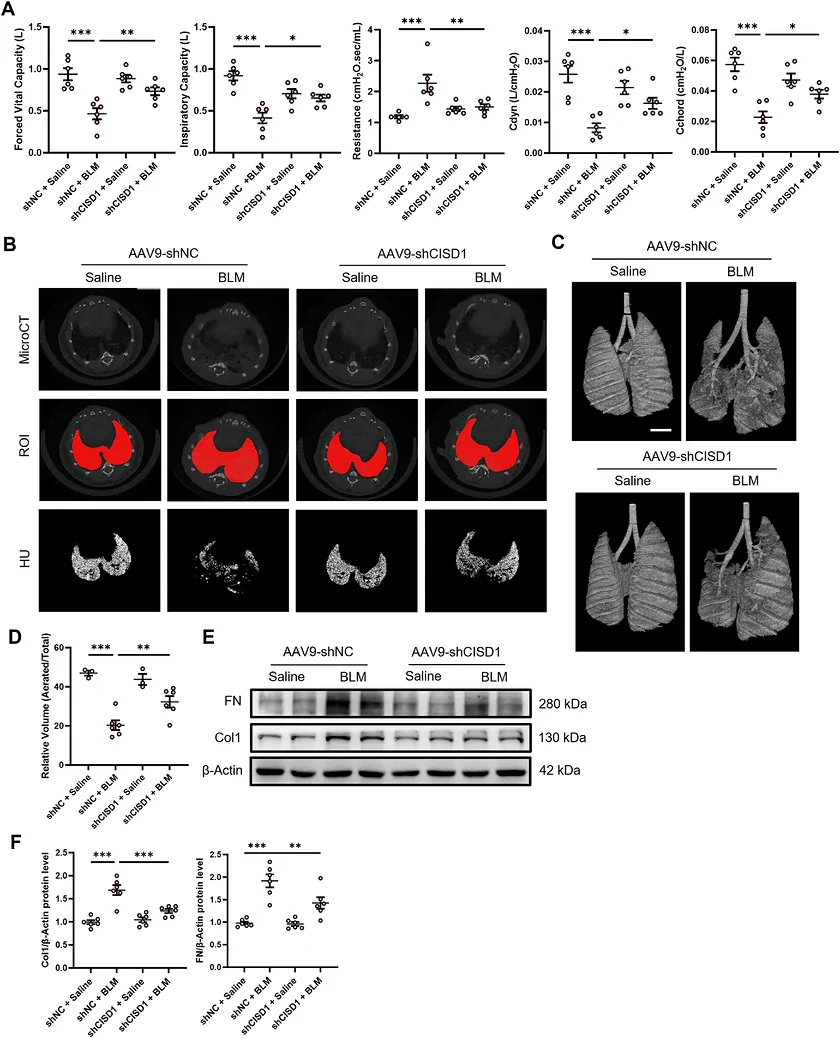
Knockdown of CISD1 ameliorates BLM‐induced pulmonary fibrosis in mice. (A) Following AAV9‐mediated knockdown of CISD1 in mice, pulmonary fibrosis was induced using BLM. The pulmonary function of mice in each group was assessed using PFT, *n* = 6 biological replicates. (B–D) MicroCT analysis of the extent of lung injury in each group of mice; 3D reconstruction of mouse lungs was performed and the ratio of normally aerated lung tissue was quantified, *n* = 3–6 biological replicates, scale bar = 2 mm. (E and F). WB detection of fibrosis‐related protein expression in lung tissues from each group of mice, *n* = 6 biological replicates.

## 3 Discussion

This study uncovers a previously unrecognized mechanotransduction axis through which the fibrotic mechanical microenvironment orchestrates macrophage‐driven inflammatory cascades, thereby driving the pathogenesis of IPF. We demonstrate that the mechanosensitive ion channel PIEZO1 is profoundly upregulated in macrophages during pulmonary fibrosis. Its activation, triggered by either elevated mechanical stress or the specific agonist Yoda1, initiates a downstream signaling cascade through the CISD1/IP3R axis. This signaling event drives cellular calcium overload, MPTP opening, and subsequent mitochondrial damage, ultimately skewing macrophages toward a pro‐inflammatory phenotype that perpetuates fibrotic progression. Notably, both genetic and pharmacological inhibition of this axis, particularly by targeting expression and activity of CISD1, effectively disrupts this pathogenic cascade, unveiling promising new therapeutic avenues for this devastating disease.

Our findings establish PIEZO1 as a pivotal upstream mechanosensor for the stiffened fibrotic niche in pulmonary fibrosis. Its observed upregulation in fibrotic lung tissue aligns well with its emerging role across multiple other fibrotic conditions [20, 27, 28, 29, 30]. While PIEZO1‐coupled mechanical cues are known to govern the function of diverse immune cells linked to disease progression [27, 31, 32], its specific functional contribution to macrophage phenotypes within IPF context has remained elusive. Interestingly, PIEZO1 activation exerts a protective effect against liver fibrosis, where targeted PIEZO1 stimulation ameliorates liver fibrosis by promoting macrophage efferocytosis [20]. Here, we posit that sustained activation of macrophage PIEZO1 in a stiff environment generates a persistent mechanical signal that disrupts intracellular calcium homeostasis. While PIEZO1‐mediated Ca^2^^+^ influx is established as the core inherent biophysical property of this channel [33], our study reveals a critical amplification mechanism that leverages ER‐dependent calcium store release to reshape downstream signaling outputs. The engagement of the CISD1/IP3R signaling axis indicates that the initial Ca^2^^+^ influx may trigger a more profound calcium release from ER stores via IP3R [22], a process consistent with well‐documented calcium‐induced calcium release (CICR) paradigm, ultimately culminating in catastrophic cellular calcium overload.

A central and novel finding from our study is the indispensable role of CISD1 in this process. As an iron‐sulfur cluster‐rich protein located on the outer mitochondrial membrane, CISD1 has been shown to regulate ER‐mitochondrial cross‐talk and lipid metabolism [22, 34]. Inhibition of CISD1 restores perturbed mitochondrial homeostasis and suppresses pathological myofibroblast activation [35]. Beyond that, CISD1 inhibition also confers protective effects against traumatic brain injury and cognitive dysfunction [36]. However, its role as a mechanistic bridge connecting mechanostress to mitochondrial pathology has not been previously documented. The precise molecular link between PIEZO1 and CISD1 has not yet been fully characterized. Previous studies have reported that mechanical stress regulates multiple fibrosis‐related genes via the Ca^2^^+^/calmodulin‐dependent protein kinase/NFAT pathway [37, 38, 39]. We therefore hypothesize that PIEZO1 transcriptionally upregulates CISD1 via the Ca^2^^+^/NFAT or Ca^2^^+^/NF‐κB signaling pathways. Further dedicated experiments are required to fully clarify this mechanism: for instance, applying specific inhibitors (the NFAT inhibitor VIVIT or NF‐κB inhibitor Bay 11–7082), or performing ChIP‐qPCR to verify the binding of target transcription factors to the CISD1 promoter. PIEZO1‐driven upregulation of CISD1 facilitates IP3R‐mediated ER Ca^2^^+^ release and directs subsequent calcium ion flux toward the mitochondria. The consequent mitochondrial calcium overload acts as a well‐established trigger for MPTP opening, inducing loss of MMP and mtROS production—all canonical signatures of PIEZO1 activation in macrophages. This previously unreported axis opens a new conceptual framework to decode the interregulation between mechanotransduction and mitochondrial effectors during pulmonary fibrosis.

While the PIEZO1/Yes‐associated protein (YAP)/transcriptional coactivator with PDZ‐binding motif (TAZ) axis is widely established as a canonical mechanotransduction cascade governing fibrosis [28, 40, 41], our data demonstrate that YAP acts in parallel to, rather than downstream of, the CISD1/IP3R axis in macrophages under fibrotic stiffness. We observed that matrix stiffness‐triggered YAP dephosphorylation (i.e., activation) was abrogated by PIEZO1 knockdown but remained unaffected by CISD1 silencing (Figure S9), suggesting that PIEZO1 activates YAP through a CISD1‐independent mechanism. This dissociation is functionally critical: PIEZO1 drives mitochondrial dysfunction and pro‐inflammatory activation through CISD1/IP3R‐mediated calcium overload, while YAP may regulate other mechano‐responsive programs including macrophage proliferation, migration, or metabolic adaptation. Notably, YAP has been functionally implicated in regulating macrophage polarization and tissue repair processes [42, 43], however, its specific role in fibrotic lung macrophages remains poorly defined. Collectively, our findings clarify that PIEZO1‐driven pro‐inflammatory mitochondrial damage is not mediated via YAP, and identify YAP and CISD1 as two bifurcating downstream arms of PIEZO1 signaling. This functional distinction holds profound implications for the design of PIEZO1‐targeted therapeutics: CISD1 inhibition would selectively abrogate mitochondrial dysfunction and inflammatory activation, while leaving YAP‐governed homeostatic or regenerative programs fully intact. This strategy could potentially deliver a far wider therapeutic safety margin than non‐selective global PIEZO1 blockade.

The significance of this PIEZO1/CISD1/IP3R‐mediated mitochondrial damage lies in its functional impact on macrophage polarization. Damaged mitochondria release potent danger‐associated molecular patterns (DAMPs) and mtROS, both of which are well‐characterized activators of the NLRP3 inflammasome and a spectrum of other pro‐inflammatory signaling cascades [17]. Moreover, PIEZO1‐mediated mechanotransduction induces calcium ion influx, activating the NLRP3 inflammasome and subsequent NLRP3‐dependent pyroptosis [31, 44]. Our prior work has established that macrophage pyroptosis promotes pulmonary fibrosis progression [17]. This uncovers a direct mechanism by which mechanical cues in the fibrotic lung program macrophages to adopt a potent pro‐inflammatory (M1‐like) phenotype. The resulting cytokine secretion recruits extra immune cells and activates collagen‐producing myofibroblasts, forming a self‐reinforcing cycle that fuels fibrosis [45].

It is noteworthy that under activation conditions, macrophages are conventionally categorized into two distinct polarized states: the classically activated pro‐inflammatory M1 phenotype and the alternately activated anti‐inflammatory/pro‐regenerative M2 phenotype, which represent the two functional extremes across the entire macrophage phenotypic spectrum. However, accumulating evidence from high‐resolution transcriptomic profiling studies strongly indicates that the conventional M1/M2 binary classification paradigm is no longer sufficient to describe the full complexity of macrophage polarization. Current consensus holds that macrophage polarization encompasses a broad range of dynamically shifting functional states, which are shaped by environmental signals, intracellular signaling cascades, and chromatin‐level regulation [46, 47]. In line with this, our animal experiments also identified the presence of CD86^+^CD206^+^ double‐positive macrophages in the lung tissue of bleomycin‐challenged mice (Figure 7B,C). This result indicates that macrophages are not simply restricted to the traditional M1 or M2 classification during pulmonary fibrosis, but can simultaneously exhibit phenotypic and functional traits of both polarization subtypes. This further demonstrates that the activation phenotype of macrophages exists as a highly plastic and dynamic state. However, it is worth noting that while our in vivo experiments reveal that macrophages exhibit both M1 and M2 phenotypes during fibrosis, macrophages cultured in vitro display an M1‐dominant phenotype. We speculate that this discrepancy arises from differences in the duration of in vitro culture. Specifically, stimulation by matrix stiffness may initially trigger M1 polarization, while sustained stimulation drives a gradual transition to the M2 phenotype. This hypothesis warrants further verification in subsequent experiments. Moreover, future investigations should incorporate more specific markers to delineate distinct activation and functional states of macrophages.

The robust efficacy of disrupting this pathway reinforces the translational promise of our findings. Inhibiting PIEZO1 activity dampens the entire downstream signaling cascade, confirming the channel's role as a critical upstream initiator. More importantly, targeting CISD1 via AAV‐mediated knockdown both in vitro and in vivo has proven remarkably effective in preserving mitochondrial integrity, suppressing inflammatory signaling, and attenuating bleomycin‐induced lung fibrosis. This positions CISD1 as a particularly compelling “bottleneck” therapeutic target. Inhibiting a downstream node such as CISD1 may confer higher specificity and a broader therapeutic window than broadly targeting a fundamental mechanosensor like PIEZO1, thereby mitigating the risk of off‐target effects.

Despite these compelling findings, limitations of our study warrant consideration. First, while we centered on macrophages, PIEZO1 is also expressed in other key fibrosis‐associated cell types, notably fibroblasts and alveolar epithelial cells. This mechanosensory pathway may function in a parallel or complementary manner across multiple cell types. Future studies employing cell‐specific knockout mouse models will be essential to delineate the relative contribution of macrophage‐specific PIEZO1/CISD1 signaling. Second, the precise molecular mechanism by which PIEZO1 activation modulates the CISD1/IP3R interaction remains to be fully elucidated. ‌Unresolved questions regarding whether this process involves direct protein–protein interactions, phosphorylation events, or transcriptional regulation of CISD1 represent exciting avenues for future investigation. In addition, the mechanical stresses examined in this study were confined to matrix stiffness (adjusted via matrix gels of varying stiffness). Beyond matrix stiffness, the fibrotic lung microenvironment also presents multiple other forms of mechanical stress—hydrostatic pressure, cyclic stretching, and fluid shear stress—all of which can be sensed by macrophages via PIEZO1 or other mechanosensitive channels. Current data do not directly address whether PIEZO1 mediates macrophage responses to these mechanical signals. Future studies using microfluidic shear devices, pressure chambers, and stretchable substrates are warranted to systematically define the role of PIEZO1 in transducing other biomechanical forces in the fibrotic lung. Of note, although our experiments validated the key molecules PIEZO1 and CISD1, in three independent models—human IPF samples, BLM‐induced mouse lung tissue, and stiff matrix‐induced macrophages—publicly available single‐cell RNA sequencing (scRNA‐seq) datasets do not show elevated transcript levels of PIEZO1 or CISD1 in macrophages (Figures S10 and S11). This highlights the heterogeneity of IPF and suggests that its regulation may occur within specific microenvironments or at the post‐transcriptional level. Finally, this study prioritized the use of male mice in the experiments to better recapitulate the primary affected population in clinical settings. As a result, the findings of this study may not be fully applicable to female mice. Given that female mice may exhibit different fibrotic responses due to sex hormone‐mediated physiological differences, future studies should incorporate experimental cohorts of female mice to assess sex‐specific phenotypic differences under identical intervention conditions.

## 4 Conclusion

We have delineated a previously unrecognized PIEZO1/CISD1/IP3R axis that mechanistically translates increased mechanical stress in the fibrotic lung into pathological macrophage activation via mitochondrial dysfunction. This pathway provides a direct link between the biophysical and biochemical drivers of IPF. Our work not only deepens the understanding of mechanobiology in fibrosis but also identifies CISD1 as a novel and promising therapeutic target for arresting the progression of pulmonary fibrosis.

## 5 Materials and Methods

### 5.1 Human Samples

The study protocol was approved by the Ethics Committee of The First Affiliated Hospital of Guangzhou Medical University (Approval No. 2018–92), and written informed consent was obtained from each participant. Patients with IPF were included in the study according to the diagnostic criteria of the 2018 ATS/ERS/JRS/ALAT clinical practice guidelines [48]. Lung tissue sections from transplanted IPF lungs or healthy donors were collected to verify PIEZO1 and CISD1 expression.

### 5.2 Animal Studies

Adult male C57/BL6J mice (8 weeks old) used in this study were purchased from Huafukang Bioscience Co., Ltd. (Beijing, China). The care and use of animals were in accordance with the guidelines of the Ethics Committee of The First Affiliated Hospital of Guangzhou Medical University (Approval No. 20250350). Mice were housed at 25°C under a 12‐h light‐dark cycle. Mice were randomly assigned to the experimental group. To induce pulmonary fibrosis, mice were anesthetized with 2% avertin and intratracheally instilled with 50 µL BLM solution (1.5 mg/kg body weight) according to our published research [1]. Control mice received an equal volume of saline. To assess the role of CISD1 in pulmonary fibrosis, mice were divided into the following groups: shNC + saline, shNC + BLM, shCISD1 + saline, and shCISD 1+ BLM. Note that 21 days post intratracheal administration of AAV9 virus (PackGene Biotech, Guangzhou, China) containing shNC or shCISD1, a BLM‐induced pulmonary fibrosis model was established, and the mice were sacrificed after an additional 21 days of housing. The sequence of mouse CISD1 shRNA is as follows: 5′‐CCGCGTAGGACCTCTGATCATCAACTCGAGTTGATGATCAGAGGTCCTACGTTTTTTG‐3′. The knockdown efficiency of CISD1 in macrophages and epithelial cells was assessed using immunofluorescence staining and western blot (Figures S8 and S12).

### 5.3 Pulmonary Function Measurements

Pulmonary function assessment was performed using an invasive pulmonary function measurement system (Buxco Research Systems, USA) in accordance with the manufacturer's operating procedures. Pulmonary function in mice was analyzed by measuring indicators such as forced vital capacity, lung resistance, inspiratory capacity, Cdyn, and Cchord.

### 5.4 MicroCT Analysis

Mice were anesthetized and transferred to a high‐resolution scanning instrument (Skyscan 1276, Bruker microCT, Germany) for scanning, following the operating guidelines provided by the manufacturer. Scan data were processed using CT Analyzer 1.16.4.1 software for noise reduction, followed by quantification of the ratio of normal aerated areas to total lung volume. Three‐dimensional reconstruction and visualization were performed using CTvox 3.3 software.

### 5.5 Histologic Analysis and Evaluation of Fibrosis

Hematoxylin and eosin (HE) staining (Solarbio, Beijing, China) was performed to assess inflammatory infiltration in lung tissues, while Masson's trichrome staining (Solarbio, Beijing, China) was applied to evaluate the extent of pulmonary fibrosis. Stained lung sections were imaged using a TissueFAXSPlus ST system (TissueGnostics, Austria) and the acquired images were quantified in ImageJ following our previously published protocol.

### 5.6 Two‐dimensional (2D) Cell Culture and Treatment

MH‐S cells derived from mice were purchased from ATCC (CRL‐2019). The cells were cultured in 1640 medium containing 10% fetal bovine serum (FBS), 100 U/mL penicillin, and 100 µg/mL streptomycin. Cells were cultured in 2D using a previously described method [25]. Briefly, transfected or untransfected MH‐S cells were seeded onto coverslips or plastic dishes coated with either 4 Kappa (soft) or 25 Kappa (stiff) polyacrylamide hydrogels plus sterile collagen, following the manufacturer's instructions. After different treatments, the cells were cultured for 24 h for subsequent experiments. The 2D ColTgel system was purchased from Bioruo (Cat: P1720, Beijing, China). The cells were transfected with siRNA (GeneReal, Guangzhou, China) and 24 h later were seeded on hydrogels of varying stiffness for culture. The specific siRNA sequence is listed in Table S1.

### 5.7 Mitochondrial Function Assay

MMP was measured using an enhanced MMP detection kit containing JC‐1 (Beyotime Biotech, Shanghai, China) according to the manufacturer's instructions. Specifically, cells were stained with 5 mM JC‐1 for 30 min at 37°C under dark conditions after different treatments. The levels of mtROS were detected using the MitoSOX staining method (Invitrogen, USA). Cells were incubated with 5 µM MitoSOX reagent working solution for 10 min at 37°C under dark conditions after different treatments. MPTP activity was detected using the MPTP assay kit (Beyotime Biotech, Shanghai, China). The results were detected using a fluorescence microscope or flow cytometer.

### 5.8 Cell Staining and Flow Cytometry

Preparation of flow cytometry staining samples followed the relevant guidelines. Before staining, cells were first blocked with anti‐mouse Fc receptor (CD16/32) antibody, followed by surface staining according to different detection panels. mtROS, membrane potential, and calcium ion concentration were detected using a flow cytometer after staining the cells according to the instructions. Flow cytometry detection was performed using FACS Verse (BD Bioscience, USA). FACS data were analyzed using FlowJo 10.8 software. The antibodies used in this study are listed in Table S2.

### 5.9 Immunofluorescence Staining

Cells were fixed with 4% paraformaldehyde, permeabilized with 0.02% Tween‐20, and blocked with goat serum. The primary antibody was diluted with blocking buffer and incubated overnight at 4°C. The secondary antibody labeled with FITC or TRITC was added and incubated for 1 h at room temperature. Cell nuclei were counterstained with DAPI‐containing mounting medium. For lung tissue immunofluorescence staining, paraffin sections were pre‐dewaxed and subjected to antigen retrieval with sodium citrate. Images were obtained under a fluorescence microscope (DM6B, Leica, Germany). Data were analyzed using ImageJ.

### 5.10 Western Blot Analysis

Lung tissue or cell protein samples were prepared using RIPA lysis buffer, and protein quantification was performed using the BCA protein assay kit (Yeasen Biotechnology, Shanghai, China). Equal amounts of protein were separated by SDS‐PAGE and transferred to PVDF membranes (Millipore, Germany). The membranes were blocked with 5% nonfat milk for 1 h at room temperature, followed by incubation with the designated antibody overnight at 4°C. The secondary antibody was added and incubated for 1 h at room temperature, and detection was performed using Tanon 5200 (Tanon, Shanghai, China). Primary antibodies are listed in Table S3.

### 5.11 RNA Extraction and Quantitative PCR Analysis

Total RNA was extracted from cells using Total RNA Extraction Reagent (Vazyme Biotech, Nanjing, China). Subsequently, the extracted RNA was reverse transcribed into cDNA with HiScript III RT SuperMix (Yeasen Biotechnology, Shanghai, China). qPCR was performed using ChamQ Universal SYBR qPCR Master Mix (Yeasen Biotechnology, Shanghai, China) to amplify target genes from each treatment group, and gene expression levels were analyzed on a real‐time PCR system (Q5 software, Applied Biosystems, USA). The 2^−ΔΔCT^ method was applied to calculate relative gene expression, with GAPDH as the internal reference. All primers were synthesized by Sangon Biotech (Shanghai, China), and the sequences are provided in Table S4.

### 5.12 Measurement of Sodium and Potassium Ions

The intracellular concentrations of sodium and potassium ions were measured using SBFI AM and PBFI AM kits (FUSHENBIO, China). Briefly, 5 mM SBFI AM or PBFI AM was mixed at a 1:1 volume ratio with Pluronic F‐127 (to facilitate dispersion into the aqueous buffer), and then diluted with an appropriate volume of aqueous buffer to a final concentration of 5 µM. The resulting probe solution was added to the cells for labeling, followed by incubation for 120 min at either room temperature or 37°C. After incubation, the cells were further incubated for 30 min in probe‐free cell loading buffer to ensure complete de‐esterification of SBFI AM/PBFI AM by the cells. Finally, the cells were washed three times with either physiological buffer or serum‐free culture medium. Fluorescence intensities were measured using a multimode microplate reader (Biotek, Winooski, VT, USA) at excitation wavelengths of 340 nm and 380 nm and an emission wavelength of 510 nm.

### 5.13 Single‐Cell RNA‐seq Data Processing and Analysis

Single‐cell RNA‐seq data from GSE122960 and GSE132771 were analyzed using the Seurat package (v4.0). Genes detected in fewer than three cells were excluded, and cells were retained according to the following quality control criteria: >200 and <7500 detected genes per cell and mitochondrial gene content <15%. Data were log‐normalized with a scale factor of 10,000, and the top 2000 highly variable genes were selected using the “vst” method. After data scaling, PCA was performed, and the first 17 principal components were retained based on the ElbowPlot. Cell clustering was conducted using an SNN graph with a resolution of 0.4, followed by UMAP visualization. Potential doublets were removed using DoubletFinder with pN = 0.25 and pK = 0.01, and batch effects were corrected using Harmony with “orig.ident” as the grouping variable. Major cell types were annotated according to canonical lineage marker genes, and macrophages were identified based on the expression of MARCO, MRC1, and MSR1. Macrophage clusters were then subsetted and re‐analyzed to assess the expression of PIEZO1 and CISD1. Differential expression between disease and HC groups was evaluated using the Wilcoxon rank‐sum test in Seurat, with Benjamini–Hochberg correction for multiple testing. UMAP plots, dot plots, and violin plots were used to visualize cell‐type annotation, macrophage marker expression, and the expression levels of PIEZO1 and CISD1 in macrophages.

### 5.14 Isolation and Culture of Murine Primary AMs

Mice were anesthetized via intraperitoneal injection of 2% Avertin and secured in a supine position. After routine disinfection of the neck region, the trachea was surgically exposed and cannulated. Bronchoalveolar lavage was performed using ice‐cold sterile physiological saline, with three sequential infusions of 0.8–1.0 mL per mouse. The recovered lavage fluid was kept on ice, pooled, then centrifuged at 300 g for 5 min at 4°C. The supernatant was discarded, and the cell pellet was resuspended in red blood cell lysis buffer, followed by a 2–3 min room‐temperature incubation to lyse residual erythrocytes. An equal volume of ice‐cold PBS was then added to terminate the lysis reaction, followed by a second centrifugation at 300 g for 5 min at 4°C. After discarding the supernatant, the cell pellet was resuspended in RPMI‐1640 complete medium supplemented with 10% (v/v) FBS. The cell suspension was seeded into culture dishes and incubated at 37°C in 5% CO_2_ for 2 h to allow adherence. Non‐adherent cells were then removed via gentle washing, and fresh medium was replenished to yield highly purified primary AMs. The purity of the enriched AM population was subsequently validated using immunofluorescence staining assays (Figure S13).

### 5.15 Statistical Analyses

Data were graphed using GraphPad Prism 10 software (GraphPad Software Inc.) and expressed as mean ± standard error of the mean (SEM). Comparisons between two groups were analyzed using unpaired two‐tailed Student's *t*‐tests, while comparisons among multiple groups (≥ 3 groups) were analyzed using one‐way analysis of variance (ANOVA) combined with Bonferroni correction. *p* values less than 0.05 were considered statistically significant.

## Author Contributions

Xiao Xiao Tang conceived, designed, and supervised the study. Yungeng Wei and Chao Xie designed specific experiments, performed experiments, and analyzed data. Xiangsheng Yang contributed to the animal study. Xiao Xiao Tang and Yungeng Wei interpreted the data and wrote the manuscript. All authors have read and approved the final manuscript.

## Funding

This study was supported by the National Natural Science Foundation of China (82470065, 82270077), R&D Program of Guangzhou National Laboratory (GZNL2025A02002), Project GZNL2025B01005 supported by Guangzhou National Laboratory and State Key Laboratory of Respiratory Disease, Innovative Drug Research and Development National Science and Technology Major Project of China (2026ZD1806500, 2026ZD1806501), the grant of State Key Laboratory of Respiratory Disease (SKLRD‐Z‐202611), and Guangzhou Institute of Respiratory Health Open Project, Guangzhou Postdoctoral Startup Fund (to Y.W.).

## Ethics Statement

The study protocol was approved by the Ethics Committee of The First Affiliated Hospital of Guangzhou Medical University (Approval No. 2018–92), and written informed consent was obtained from each participant. The care and use of animals were in accordance with the guidelines of the Ethics Committee of The First Affiliated Hospital of Guangzhou Medical University (Approval No. 20250350).

## Conflicts of Interest

The authors declare no conflicts of interest.

## Supporting information




**Supporting File**: mco271080‐sup‐0001‐SuppMat.docx.

## Data Availability

The data that support the findings of this study are presented within the article and the Supplementary files.
