# Supplementary material for: The PIEZO1/CISD1/IP3R Mechanotransduction Axis Drives Macrophage Mitochondrial Dysfunction and Inflammatory Activation in Pulmonary Fibrosis
Source: MedComm (2020). 2026 Oct 15;7(11):e71080. doi: 10.1002/mco2.71080 (PMC13670015; doi:10.1002/mco2.71080)
Supplement: Supplementary file 1 [file MCO2-7-e71080-s001.docx]

**The PIEZO1/CISD1/IP3R Mechanotransduction Axis Drives Macrophage Mitochondrial Dysfunction and Inflammatory Activation in Pulmonary Fibrosis**

**Running title: THE PIEZO1-CISD1 AXIS IN PULMONARY FIBROSIS**

Yungeng Wei^#1^, Chao Xie^#1^, Xiangsheng Yang^1^, Xiao Xiao Tang^1,2,3^*.

1 State Key Laboratory of Respiratory Disease, National Clinical Research Center for Respiratory Disease, National Center for Respiratory Medicine, Guangzhou Institute of Respiratory Health, The First Affiliated Hospital of Guangzhou Medical University, Guangzhou, China

2 Guangzhou National Laboratory, Bio-island, Guangzhou, China

3 GMU-GIBH Joint School of Life Sciences, Guangzhou Medical University, Guangzhou, China

*Correspondence:

Prof. Xiao Xiao Tang, Email: tangxiaoxiao@gird.cn

ORCID: 0000-0002-3383-0989

^#^ Contributed equally

**Supporting Information**

**Supplementary Tables**

**Table S1. Sequence with gene knockdown**

| Gene | Sense (5’-3’) | Anti-sense (5’-3’) |
| --- | --- | --- |
| *Mus-siPiezo1* | AGGAGAAGCUGCUGAAGAATT | UUCUUCAGCAGCUUCUCCUTT |

**Table S2. List of anti-mouse antibodies used in flow cytometry**

| Target | Conjugate | Clone |
| --- | --- | --- |
| CD45 | APC/Cy7 | 30-F11, Biolegend |
| CD11b | Percp/Cy5.5 | M1/70, BD Bioscience |
| CD11c | PE/Cy7 | HL3, BD Bioscience |
| F4/80 | PE | BM8, Biolegend |
| F4/80 | BV421 | T45-2342 (RUO), BD Bioscience |
| CD206 | BV421 | C068C2, Biolegend |
| CD86 | BV510 | GL-1, Biolegend |

**Table S3. List of antibodies used in WB analysis**

| Target | Cat. No. |
| --- | --- |
| PIEZO1 | 15939-1-AP, Proteintech |
| CypD | ab110324, Abcam |
| ANT1 | ab99292, Abcam |
| CD163 | WL03026, Wanleibio |
| iNOS | 18985-1-AP, Proteintech |
| NLRP3 | ab263899, Abcam |
| CISD1 | 16006-1-AP, Proteintech |
| IP3R | ab108517, Abcam |
| MCU | A16281, ABclonal |
| FN | sc-8422, Santa Cruz |
| Col1 | 14695-1-AP, Proteintech |
| β-Actin | 8457, CST |

**Table S4. Primer sequences for qPCR in this study**

| Gene | Forward (5’-3’) | Reverse (5’-3’) |
| --- | --- | --- |
| *Mus-Gapdh* | AGGTCGGTGTGAACGGATTTG | TGTAGACCATGTAGTTGAGGTCA |
| *Mus-Nos2* | GTTCTCAGCCCAACAATACAAGA | GTGGACGGGTCGATGTCAC |
| *Mus-Il1b* | GCAACTGTTCCTGAACTCAACT | ATCTTTTGGGGTCCGTCAACT |
| *Mus-Il6* | TAGTCCTTCCTACCCCAATTTCC | TTGGTCCTTAGCCACTCCTTC |
| *Mus-Tnfa* | CCCTCACACTCAGATCATCTTCT | GCTACGACGTGGGCTACAG |
| *Mus-Mrc1* | CTCTGTTCAGCTATTGGACGC | CGGAATTTCTGGGATTCAGCTTC |
| *Mus-Arg1* | CTCCAAGCCAAAGTCCTTAGAG | AGGAGCTGTCATTAGGGACATC |
| *Mus-Il10* | GCTCTTACTGACTGGCATGAG | CGCAGCTCTAGGAGCATGTG |
| *Mus-Ant1* | GAGGCGTGGATCGCCATAAG | CACTTGGGGGAGTTCATGCT |
| *Mus-CypD* | CTTCCACAGGGTGATCCCAG | ACTGAGAGCCATTGGTGTTGG |

**Supplementary Figures**

**Figure S1**

MH-S cells were cultured on a stiff matrix for 24 hours, after which the concentrations of sodium and potassium ions were measured. Compared with the 4 KPa group, the concentration of K+ decreased in the 25 KPa group, while the Na+ concentration increased. These results indicate that substrate stiffness elevation-mediated PIEZO1 activation leads to alterations in intracellular Na+ and K+ homeostasis.

**Figure S2**

A. MH-S cells were incubated with matrix gels of different stiffness for 24 h. Flow cytometry analysis of macrophage polarization based on CD86 (M1) and CD206 (M2) expression, data shown as Mean ± SEM, n=4 biological replicates.

B. MH-S cells were incubated with matrix gels of different stiffness for 24 h. qPCR analysis of the expression of M1 macrophage markers Nos2, Il1b, Il6, and Tnfa, as well as M2 macrophage markers Arg1, Mrc1, and Il10, data shown as Mean ± SEM, n=6 biological replicates.

**Figure S3**

**Inhibition of CISD1 attenuates mechanical stiffness-mediated macrophage MPTP opening and mitochondrial damage.**

A. MH-S cells were cultured with different stiffness matrix gels and simultaneously treated with CISD1 inhibitor NL-1, calcium ion concentration in cytoplasm was detected by flow cytometry, n=5 biological replicates.

B. MH-S cells were cultured with different stiffness matrix gels and simultaneously treated with CISD1 inhibitor NL-1, and MPTP activity was detected by flow cytometry, n=5 biological replicates.

C. MH-S cells were cultured with different stiffness matrix gels and simultaneously treated with CISD1 inhibitor NL-1, mitochondrial reactive oxygen species content was detected by flow cytometry, n=5 biological replicates.

D-E. MH-S cells were cultured with different stiffness matrix gels and simultaneously treated with CISD1 inhibitor NL-1, mitochondrial membrane potential was detected by immunofluorescence microscope, n=3 biological replicates, scale bar=20 μm.

F-G. MH-S cells were cultured with different stiffness matrix gels and simultaneously treated with CISD1 inhibitor NL-1, MPTP-related proteins and inflammation-related proteins were detected by WB, n=5 biological replicates.

**Figure S4**

MH-S cells were treated with 5 μM Yoda1 for 24h, cytosolic calcium ions were measured by flow cytometry, data shown as Mean ± SEM, n=3 biological replicates.

**Figure S5**

**Matrix stiffness upregulates CISD1 via PIEZO1 to mediate mitochondrial damage.**

A. MH-S cells were cultured with matrix gels of different stiffness and simultaneously treated with PIEZO1 inhibitor GsMTx4, WB assay was performed to detect the expression of MPTP-related proteins, n=4 biological replicates.

B. MH-S cells were cultured with different stiffness matrix gels and simultaneously treated with PIEZO1 inhibitor GsMTx4, and MPTP activity was detected by flow cytometry, n=3 biological replicates.

C. MH-S cells were cultured with different stiffness matrix gels and simultaneously treated with PIEZO1 inhibitor GsMTx4, mitochondrial reactive oxygen species content was detected by flow cytometry, n=5 biological replicates.

D. MH-S cells were cultured with different stiffness matrix gels and simultaneously treated with PIEZO1 inhibitor GsMTx4, mitochondrial membrane potential was detected by immunofluorescence microscopy, n=4 biological replicates.

**Figure S6**

**Knockdown of PIEZO1 alleviates matrix stiffness-induced mitochondrial damage.**

A. MH-S cells with knockdown of PIEZO1 were cultured on different stiffness matrix gels, WB assay was performed to detect the expression of MPTP-related proteins, n=4 biological replicates.

B. MH-S cells with knockdown of PIEZO1 were cultured on different stiffness matrix gels, and MPTP activity was detected by flow cytometry, n=5 biological replicates.

C. MH-S cells with knockdown of PIEZO1 were cultured on different stiffness matrix gels, mitochondrial reactive oxygen species content was detected by flow cytometry, n=5 biological replicates.

D. MH-S cells with knockdown of PIEZO1 were cultured on different stiffness matrix gels, mitochondrial membrane potential was detected by immunofluorescence microscopy, n=3 biological replicates, scale bar=20 μm.

**Figure S7**

**Knockdown of CISD1 ameliorates BLM-induced pulmonary fibrosis in mice.**

A. HE staining analysis of inflammatory infiltration in lung sections from each group of mice, n=6 biological replicates, scale bar=1 mm (top) and 50 μm (bottom).

B. Masson's trichrome staining analysis of collagen deposition in lung sections from each group of mice, n=6 biological replicates, scale bar=1 mm (top) and 50 μm (bottom).

C. Immunofluorescence staining was used to detect α-SMA expression in the lungs of mice in each group, n=6 biological replicates, scale bar= 20 μm.

**Figure S8**

A. The efficiency of CISD1 knockdown in lung tissue macrophages by adeno-associated virus (AAV) was assessed using immunofluorescence staining. Co-localization of the macrophage marker CD68 with CISD1 was examined. AAV9-shCISD1 effectively knocked down BLM-induced CISD1 expression in macrophages. Scale bar = 20 µm.

B. WB detection of CISD1 protein expression in lung tissues from each group of mice, n=6 biological replicates.

**Figure S9**

A. WB analysis of PIEZO1 knockdown in MH-S cells cultured on a stiff matrix gel revealed YAP activation, data shown as Mean ± SEM, n=3 biological replicates.

B. WB analysis of CISD1 knockdown in MH-S cells cultured on a stiff matrix gel revealed YAP activation, data shown as Mean ± SEM, n=3 biological replicates.

**Figure S10**

**Single-cell RNA-seq profiling of GSE122960.**

A-B. UMAP plot showing all clusters and celltypes.

C. Dotplot showing representative markers for each cluster.

D-F. UMAP plots showing the expression of macrophage marker genes MARCO, MRC1, and MSR1.

G. Violin plots showing the expression of PIEZO1 across macrophage subtypes.

H. Violin plots showing the expression of CISD1 across macrophage subtypes.

**Figure S11**

**Single-cell RNA-seq profiling of GSE132771.**

A-B. UMAP plot showing all clusters and celltypes.

C. Dotplot showing representative markers for each cluster.

D-F. UMAP plots showing the expression of macrophage marker genes MARCO, MRC1, and MSR1.

G. Violin plots showing the expression of PIEZO1 across macrophage subtypes.

H. Violin plots showing the expression of CISD1 across macrophage subtypes.

**Figure S12**

Immunofluorescence staining was performed to evaluate the efficiency of AAV-mediated CISD1 knockdown in lung tissue epithelial cells and to examine the co-localization of CISD1 with the epithelial cell marker SPC. Although AAV9-shCISD1 effectively knocked down BLM-induced CISD1 expression, CISD1 was not predominantly expressed in epithelial cells. Scale bar = 20 µm.

**Figure S13**

Primary cells were isolated from mouse bronchoalveolar lavage fluid and further purified via adherent culture. The resulting cells were immunostained with an anti-F4/80 antibody (red), while cell nuclei were counterstained with DAPI (blue). Subsequent imaging analysis revealed that nearly all adherent cells were F4/80-positive, confirming the high purity of the isolated alveolar macrophage population. Scale bar = 50 μm.
